# Propensity-matched analysis of robotic versus sternotomy approaches for mitral valve replacement

**DOI:** 10.1007/s11701-023-01665-0

**Published:** 2023-07-09

**Authors:** Wenlong Yan, Yangyang Wang, Wei Wang, Qingjiang Wang, Xin Zheng, Sumin Yang

**Affiliations:** 1 Department of Cardiovascular Surgery, The Affiliated Hospital of Qingdao University, Qingdao, Shandong China; 2 Department of Nuclear Medicine, The Affiliated Hospital of Qingdao University, Qingdao, Shandong China; 3 Surgical Operating Room, The Affiliated Hospital of Qingdao University, Qingdao, Shandong China

**Keywords:** Mitral valve replacement, Robot, Sternotomy, Propensity score matching

## Abstract

To compare early and medium-term outcomes between robotic and sternotomy approaches for mitral valve replacement (MVR). Clinical data of 1393 cases who underwent MVR between January 2014 and January 2023 were collected and stratified into robotic MVR (n = 186) and conventional sternotomy MVR (n = 1207) groups. The baseline data of the two groups of patients were corrected by the propensity score matching (PSM) method. After matching, the baseline characteristics were not significant different between the two groups (standardized mean difference < 10%). Moreover, the rates of operative mortality (P = 0.663), permanent stroke (P = 0.914), renal failure (P = 0.758), pneumonia (P = 0.722), and reoperation (P = 0.509) were not significantly different. Operation, CPB and cross-clamp time were shorter in the sternotomy group. On the other hand, ICU stay time, post-operative LOS, intraoperative transfusion, and intraoperative blood loss were shorter or less in the robot group. Operation, CPB, and cross-clamp time in robot group were all remarkably improved with experience. Finally, all-cause mortality (P = 0.633), redo mitral valve surgery (P = 0.739), and valve-related complications (P = 0.866) in 5 years of follow-up were not different between the two groups. Robotic MVR is safe, feasible, and reproducible for carefully selected patients with good operative outcomes and medium-term clinical outcomes.

## Introduction

Mitral valve (MV) disease is one of the commonest valvular cardiac disorders worldwide [1]. Common types of mitral valve disease in adults include degenerative, rheumatic, ischemic and infectious processes. The treatment of these different mitral valve diseases depends on the cause, pathophysiology, and natural history of each disease. According to current guidelines [2, 3], mitral valve repair (MVP) and percutaneous mitral balloon commissurotomy (PMBC) are the preferred treatment for mitral regurgitation and mitral stenosis. However, mitral valve replacement (MVR) remains an important treatment option for patients not eligible for MVR or PMBC, especially in rheumatic MV cases.

With the decrease in operative mortality and postoperative complications, patients put forward new and higher requirements for mitral valve surgery. For example, the sternotomy is often associated with long recovery times, poor cosmetic results, and severe deep sternal wound infection. To avoid some drawbacks of sternotomy, the minimally invasive MV technique first appeared in the 1990s and has since gained popularity [4]. After nearly three decades of development, robotic systems now have excellent three-dimensional views, precise movements, and auxiliary equipment that can shorten the learning cycle for surgeons [5]. The feasibility and safety of robotic MVP has been proved in many studies [6–9], with the primary goal of improving cosmetic results and reducing postoperative complications while maintaining the same prognosis as sternotomy surgery. However, the reports comparing robotic and sternotomy MVR are limited. Thus, the current investigation was developed to report the early and medium-term outcomes of robotic MVR in comparison with sternotomy approach.

## Patients and methods

### Study population

**Fig. 1:**
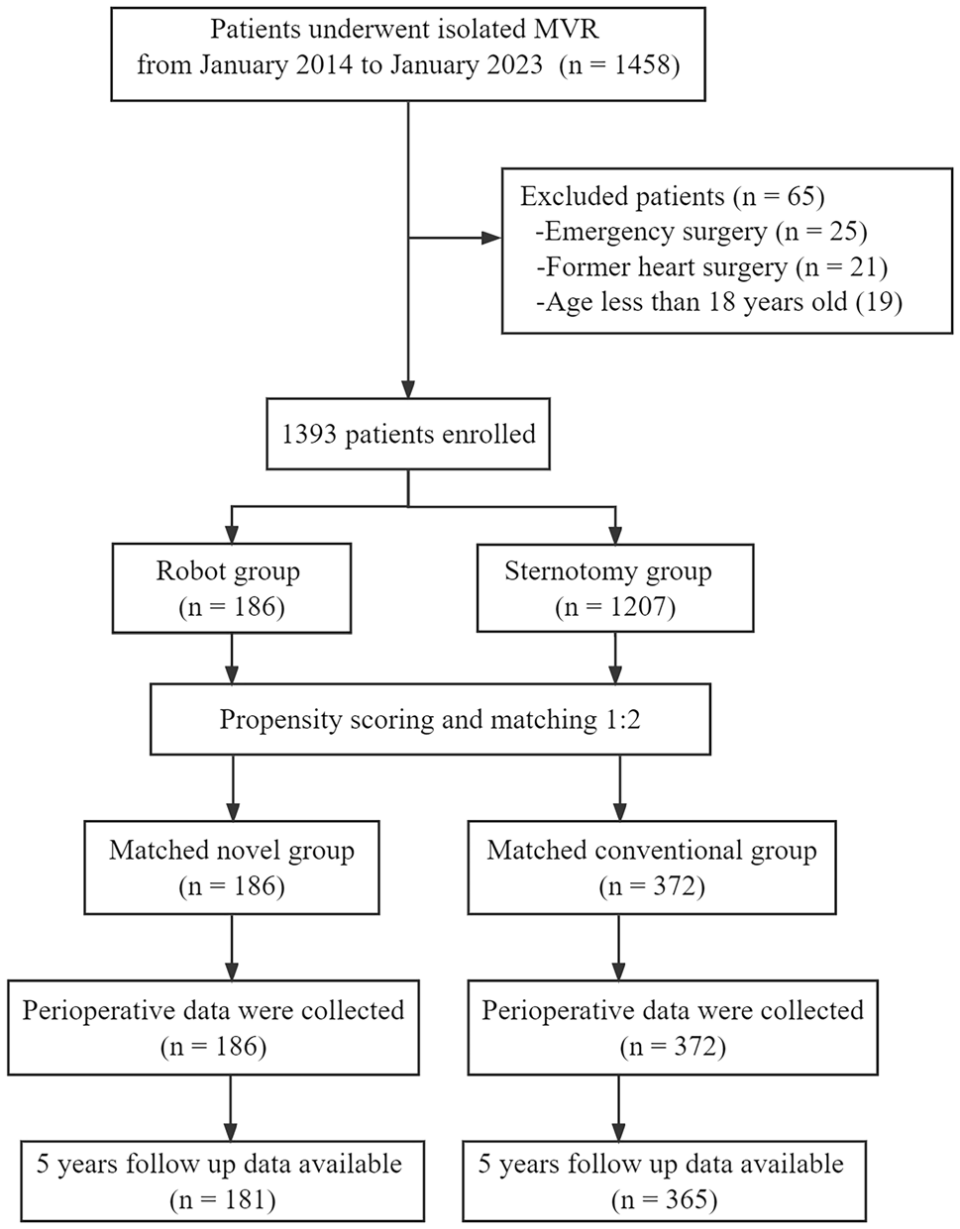
Patient flow diagram

This retrospective analysis was conducted on 1458 patients diagnosed with MV disease who underwent MVR surgery at the Affiliated Hospital of Qingdao University from January 2014 to January 2023 (Fig. 1). Patients in this study met the recommendations of 2020 ACC/AHA (United States) or 2021 ESC/EACTS (European) guidelines [2, 3]. The exclusion criteria included: patients who received emergency surgery, former heart surgery and less than 18 years old. And patients who received MVR combined with other surgery (except tricuspid repair, radiofrequency ablation and thrombectomy) were also excluded. This study included the patient who initially planned MVP and converted to MVR. The included participants were stratified into two groups, the robot and sternotomy groups based on the approach. Patients selected for robotic surgery were carefully screened, and the exclusion criteria for robotic MVR are shown in Table 1. The safety and clinical efficacy of the two surgical methods were compared using the propensity score matching (PSM) method to decrease differences and bias that might influence the treatment selection and outcomes. The screening algorithm used for patients with MV disease is depicted in Fig. 1. This study was approved by the Institutional Review Board of the Affiliated Hospital of Qingdao University (approval number: QYFY WZLL 27718; date: March 29, 2023), and the requirement for informed consent was waived.

**Table 1:** Exclusion criteria for robotic surgery

Severe pleural adhesions (previous right thoracotomy, thoracic trauma, pleuritis)
Severe pulmonary dysfunction
LV dysfunction
Pulmonary artery pressure > 70 mm Hg or severe RV dysfunction
LV dysfunction
Femoral artery diameter < 7 mm
Severe peripheral vascular disease
Renal disease
Significant mitral annular calcification
Kyphoscoliosis and pectus excavatum
Morbid obesity
Greater than mild aortic regurgitation or significant aortic stenosis
Coronary artery disease—requiring CABG

*LV* left ventricle, *RV* right ventricle, *CABG* coronary artery bypass grafting

### Surgical methods

**Fig. 2:**
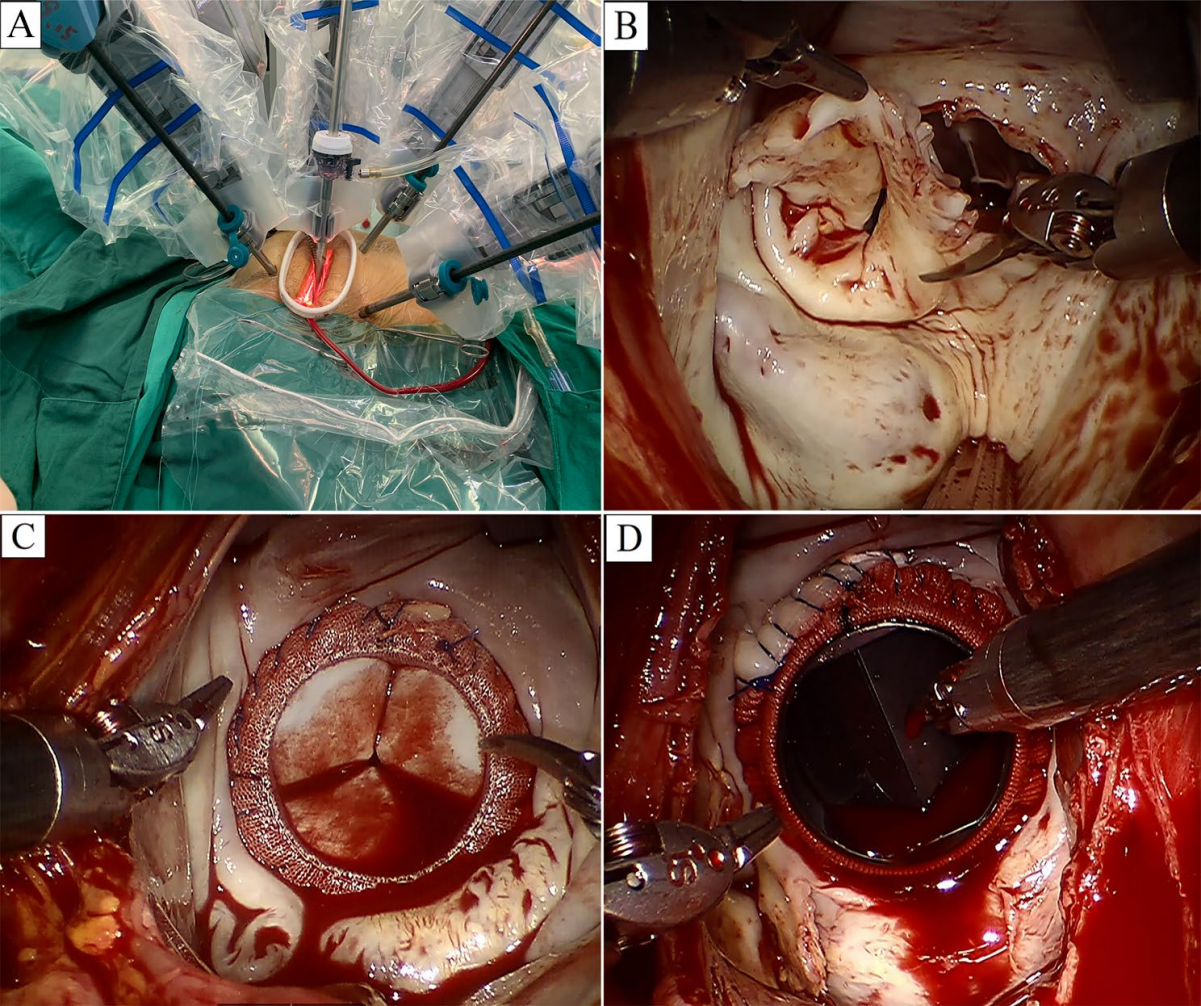
Robotic mitral valve replacement. **A** Four robotic arms are docked to a patient’s right chest. From left to right: left instrument arm, camera arm, dynamic retractor arm, and right instrument arm. **B** The diseased mitral valve is excised. **C** Robotically seated bioprosthetic valve. **D** Robotically seated mechanical valve

Robotic surgery was performed with the daVinci Surgical System SI (Intuitive Surgical, Inc., Mountain View, CA, USA). After inducing general anesthesia, isolating the right lung was essential, for which a right-sided bronchial blocker or a double-lumen endotracheal tube were approached, being the commonest modalities in this context. External defibrillator pads were applied, crossing the cardiac mass, and connected before starting the procedure. Then a transesophageal echocardiography (TEE) probe, right internal jugular venous drainage cannula, and Swan-Ganz catheter were applied. Normal placement has the patient lying supine having their right thorax raised and the right arm at the side, which causes the shoulder to remain moved backwards. A 23-Fr or 21-Fr cannula (Medtronic, Minneapolis, MN, USA) is inserted into the right femoral vein, and a 15-Fr cannula (Medtronic) is inserted into the right internal jugular vein to facilitate venous drainage. Using a 20-Fr or 18-Fr cannula (Medtronic, Minneapolis, MN, USA), retrograde arterial blood flow is created via the right femoral artery. In the right 4th intercostal space, laterally to the anterior axillary line, a working port of 4–5 cm is created. In the right 2nd intercostal space, on the anterior axillary line, is where the port for the left robot arm was implanted. The port for the right robot arm was situated in the right 6th intercostal space, midaxillary line. The mid-clavicular line of the right 5th intercostal space was chosen as the ideal location for the dynamic retractor arm (Fig. 2A). The Chitwood Transthoracic Aortic Cross-clamp (Scanlan International, Minneapolis, MN) was the choice when cross-clamping the aorta through the chest. Repeated doses of antegrade cold blood cardioplegic solution were delivered via the working port when needed. To avoid camera fogging and to remove air from the hemithorax, warm CO_2_ was continually insufflated into the operating area. As seen in Fig. 2B, the posterior leaflets were retained wherever feasible during the removal of the sick mitral valve. An incision was made in the left atrium perpendicular to the atrial septum. A dynamic atrial retractor was used to reveal the mitral valve. In order to have a clean surgical site, a flexible drainage catheter was inserted into the left superior pulmonary vein. Carbo-Medics mechanical valve (Sulzer Carbomedics, Austin, TX), St. Jude Medical Regent Mechanical Heart Valve (St. Paul, MN), and Carpentier-Edwards PERIMOUNT Plus Pericardial Bioprosthesis (Edwards Lifesciences, Irving, CA) were the replacement valves (Fig. 2C, D). After the sutures were threaded through the incision and inserted in the prosthesis sewing ring outside of the chest, the prosthesis was dropped into place and the knots were tied with the help of a knot pusher. De-airing the heart, closing the atrium, and removing cardiopulmonary bypass allowed for a thorough TEE examination. When the right atrium must be opened for a concomitant tricuspid valve repair, caval tapes are applied to prevent air entrainment.

### Statistical analysis

Student’s t-test was used to examine normally scattered continuous data represented as mean standard deviation (SD). Median and interquartile ranges were calculated for continuous data with no normal distribution, and the Mann–Whitney U test was used for statistical significance. Furthermore, Fisher’s exact test or Pearson’s χ^2^ test was used to examine data using categorical variables, which were reported as percentages. The data was analyzed considering all patients who were eligible to participate. To minimize bias between the two groups, PSM was calculated using a logistic regression based on eighteen factors: age, male, body mass index (BMI), hypertension, diabetes mellitus, peripheral arterial disease (PAD), renal disease, chronic lung disease, cerebrovascular disease, New York Heart Association (NYHA) class, left ventricular ejection fraction (LVEF), left ventricular end–diastolic diameter (LVEDD), left atrial anterior and posterior diameter, systolic pulmonary artery pressure (SPAP), cardiothoracic ratio (CTR), Etiology, concomitant surgery, Society of Thoracic Surgeons predicted risk of mortality (STS PROM). Standardized mean differences (SMD) were used to assess the equilibrium after matching, and PSM was carried out using the closest neighbor approach with a 0.2 caliper and a 1:2 ratio. Operation time, CPB time and cross-clamp time were analyzed using a non-linear regression model (r^2^) to assess learning period effects. The hazard ratio (HR) of clinical outcomes was determined using the Cox proportional hazard model, and the time-to-event Kaplan–Meier curve was used to show the progression of all-cause mortality, subsequent mitral valve surgery, and valve-related comorbidities. The PSM analysis was conducted using R Analysis 4.2.2 (R Core Team, R Foundation for Statistical Computing, Vienna, Austria). SPSS version 23.0 (IBM, Armonk, NY, USA) was used to run other statistical analyses and generate the graphics. Statistical significance was assumed when the P value was less than 0.05.

## Results

### Baseline characteristics

**Fig. 3:**
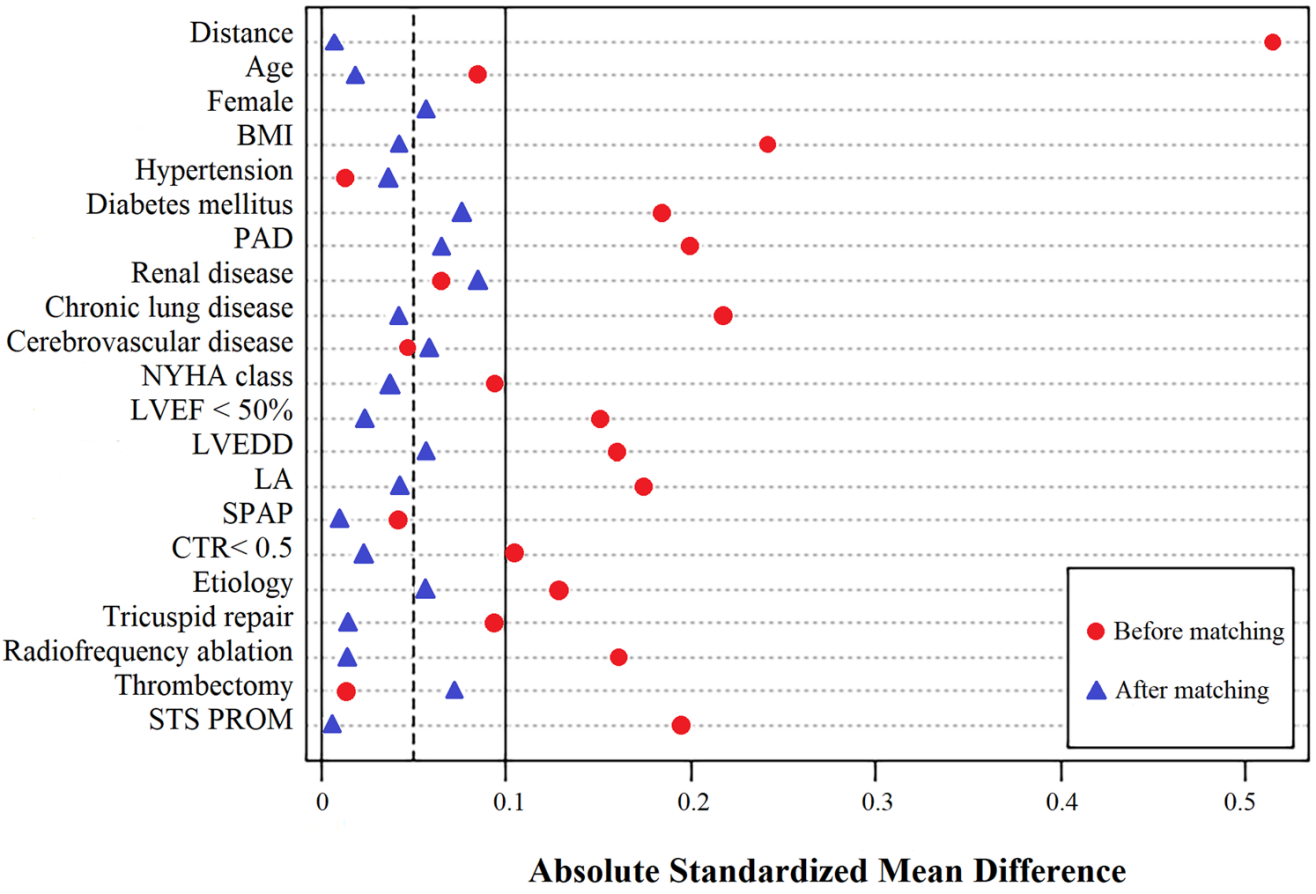
Absolute standardized mean differences

Totally, 1458 procedures were conducted, of which 25, 21, and 19 cases were excluded being emergency, with a history of cardiac operation, and conducted with age < 18 years. Eventually, 1393 cases were included. Rheumatic MV disease was the commonest condition in this population. Patients were grouped into robot (N = 186), and sternotomy (N = 1207) groups. Different baseline characteristics are listed in Table 2. The two groups had a statistically significant difference in BMI, chronic lung disease, LVEDD, LA, and STS PROM. Furthermore, 558 cases were considered after PSM with a standardized mean difference (SMD) of < 10% for all variables (Table 3). The absolute SMD before and after matching are shown in Fig. 3.

**Table 2:** Baseline characteristics before matching

	Robot group (n = 186)	Sternotomy group (n = 1207)	P value
Age (years)	61.4 ± 8.8	62.2 ± 8.3	0.225
Female	121 (65.1%)	818 (67.8%)	0.462
BMI (kg/m^2^)	23.6 ± 3.1	24.4 ± 3.6	0.004
Hypertension	31 (16.7%)	207 (17.1%)	0.870
Diabetes mellitus	21 (11.3%)	149 (12.3%)	0.683
PAD	3 (1.6%)	50 (4.1%)	0.093
Renal disease	5 (2.7%)	45 (3.7%)	0.478
Chronic lung disease	8 (4.3%)	105 (8.7%)	0.041
Cerebrovascular disease	6 (3.2%)	49 (4.1%)	0.587
NYHA class			0.684
I	17 (9.1%)	97 (8.0%)	
II	97 (52.2%)	587 (48.6%)	
III	67 (36.0%)	483 (40.0%)	
IV	5 (2.7%)	40 (3.3%)	
LVEF < 50%	10 (5.4%)	106 (8.8%)	0.118
LVEDD, mm	45.7 ± 6.8	46.9 ± 6.3	0.017
LA, mm	48.3 ± 8.5	49.9 ± 8.8	0.021
SPAP	45.1 ± 13.8	45.7 ± 14.2	0.590
CTR < 0.5	102 (54.8%)	601 (49.8%)	0.200
Etiology			0.294
Rheumatic	113 (60.8%)	667 (55.3%)	
Degenerative	68 (36.6%)	489 (40.5%)	
Other	5 (2.7%)	51 (4.2%)	
Concomitant surgery			
Tricuspid repair	34 (18.3%)	264 (21.9%)	0.266
Radiofrequency ablation	38 (20.4%)	325 (26.9%)	0.060
Thrombectomy	18 (9.7%)	121 (10.0)	0.883
STS PROM (%)	4.8 ± 1.7	5.1 ± 1.6	0.018

*BMI* body mass index, *PAD* peripheral arterial disease, *NYHA* New York Heart Association, *LVEF* left ventricular ejection fraction, *LVEDD* left ventricular end–diastolic diameter, *LA* left atrium, *SPAP* systolic pulmonary artery pressure, *CTR* cardiothoracic ratio, *STS* Society of Thoracic Surgeons, *PROM* predicted risk of mortality

**Table 3:** Baseline characteristics after matching

	Robot group (n = 186)	Sternotomy group (n = 372)	P value	Absolute SMD
Age (years)	61.4 ± 8.8	61.2 ± 8.5	0.796	0.017
Female	121 (65.1%)	252 (67.7%)	0.525	0.056
BMI (kg/m^2^)	23.6 ± 3.1	23.5 ± 3.3	0.731	0.042
Hypertension	31 (16.7%)	64 (17.2%)	0.873	0.036
Diabetes mellitus	21 (11.3%)	51 (13.7%)	0.422	0.076
PAD	3 (1.6%)	9 (2.4%)	0.757	0.064
Renal disease	5 (2.7%)	12 (3.2%)	0.728	0.083
Chronic lung disease	8 (4.3%)	18 (4.8%)	0.776	0.039
Cerebrovascular disease	6 (3.2%)	11 (3.0%)	0.862	0.061
NYHA class			0.963	0.035
I	17 (9.1%)	31 (8.3%)		
II	97 (52.2%)	189 (50.8%)		
III	67 (36.0%)	141 (37.9%)		
IV	5 (2.7%)	11 (3.0%)		
LVEF < 50%	10 (5.4%)	22 (5.9%)	0.797	0.024
LVEDD, mm	45.7 ± 6.8	45.4 ± 6.6	0.617	0.057
LA, mm	48.3 ± 8.5	48.7 ± 8.9	0.612	0.044
SPAP	45.1 ± 13.8	45.0 ± 13.6	0.590	0.010
CTR < 0.5	102 (54.8%)	208 (55.9%)	0.810	0.022
Etiology			0.909	0.054
Rheumatic	113 (60.8%)	219 (58.9%)		
Degenerative	68 (36.6%)	142 (38.2%)		
Other	5 (2.7%)	11 (3.0%)		
Concomitant surgery				
Tricuspid repair	34 (18.3%)	66 (17.7%)	0.876	0.014
Radiofrequency ablation	38 (20.4%)	74 (19.9%)	0.881	0.013
Thrombectomy	18 (9.7%)	40 (10.8)	0.695	0.073
STS PROM (%)	4.8 ± 1.7	4.7 ± 1.8	0.529	0.003

*SMD* standardized mean difference, *BMI* body mass index, *PAD* peripheral arterial disease, *NYHA* New York Heart Association, *LVEF* left ventricular ejection fraction, *LVEDD* left ventricular end–diastolic diameter, *LA* left atrium, *SPAP* systolic pulmonary artery pressure, *CTR* cardiothoracic ratio, *STS* Society of Thoracic Surgeons, *PROM* predicted risk of mortality

### Perioperative data

In the robot group, all 186 patients underwent successful robotic MVR with bioprosthetic or mechanical valves. The function of prostheses was confirmed satisfactory by intraoperative TEE in all patients. Perioperative data before matching are presented in Table 4. Cross-clamp, CPB, and operation times were all significantly shorter in the sternotomy group, whereas ICU stay time, post-operative LOS, intraoperative transfusion, postoperative transfusion and intraoperative blood loss were significantly shorter or less in the robot group. Operative mortality before matching was significantly lower in the robot group than in the sternotomy group. Perioperative data after matching are presented in Table 5. Operation, CPB and cross-clamp times were still shorter in the sternotomy group. ICU stay time, post-operative LOS, intraoperative transfusion and intraoperative blood loss were still shorter or less in the robot group. Operative mortality and postoperative transfusion became no difference between the two groups.

**Table 4:** Perioperative outcomes before matching

	Robot group (n = 186)	Sternotomy group (n = 1207)	P value
Bioprosthesis implantation	111 (59.7%)	728 (60.3%)	0.869
Operation time, min	237 ± 51	188 ± 43	< 0.001
CPB time, min	155 ± 42	112 ± 25	< 0.001
Cross-clamp time	98 ± 31	80 ± 21	< 0.001
ICU stay time, hour	27.3 ± 7.6	34.2 ± 8.1	< 0.001
Post-operative LOS, day	9.6 ± 3.3	11.2 ± 3.8	< 0.001
Intraoperative transfusion, units	1.1 ± 0.8	1.8 ± 1.1	< 0.001
Postoperative transfusion, units	2.3 ± 1.7	3.0 ± 1.5	< 0.001
Intraoperative blood loss, mL	435 ± 188	520 ± 212	< 0.001
Operative mortality	1 (0.5%)	40 (3.3%)	0.037
Reoperation	5 (2.7%)	55 (4.6%)	0.243
Wound infection	2 (1.1%)	21 (1.7%)	0.724
Permanent stroke	3 (1.6%)	36 (3.0%)	0.292
Permanent ventilation > 24 h	29 (15.6%)	175 (14.5%)	0.695
Renal failure	8 (4.3%)	57 (4.7%)	0.800
Permanent pacemaker implantation	4 (2.1%)	29 (2.4%)	0.961
Pneumonia	4 (2.2%)	22 (1.8%)	0.986
New onset atrial fibrillation	45 (24.2%)	352 (29.2%)	0.162
IABP	5 (2.7%)	49 (4.1%)	0.367
ECMO	2 (1.1%)	20 (1.7%)	0.782

*CPB* cardiopulmonary bypass, *ICU* intensive care unit, *LOS* length of stay, *IABP* intra-aortic balloon pump, *ECMO* extracorporeal membrane oxygenation

**Table 5:** Perioperative outcomes after matching

	Robot group (n = 186)	Sternotomy group (n = 372)	P value
Bioprosthesis implantation	111 (59.7%)	208 (55.9%)	0.397
Operation time, min	237 ± 51	179 ± 39	< 0.001
CPB time, min	155 ± 42	106 ± 21	< 0.001
Cross-clamp time	98 ± 31	78 ± 18	< 0.001
ICU stay time, hour	27.3 ± 7.6	28.9 ± 7.9	0.023
Post-operative LOS, day	9.6 ± 3.3	10.4 ± 3.6	0.011
Intraoperative transfusion, units	1.1 ± 0.8	1.3 ± 0.7	0.003
Postoperative transfusion, units	2.3 ± 1.7	2.5 ± 1.6	0.173
Intraoperative blood loss, mL	435 ± 188	478 ± 191	0.012
Operative mortality	1 (0.5%)	5 (1.3%)	0.663
Reoperation	5 (2.7%)	14 (3.8%)	0.509
Wound infection	2 (1.1%)	6 (1.6%)	0.900
Permanent stroke	3 (1.6%)	8 (2.2%)	0.914
Permanent ventilation > 24 h	29 (15.6%)	49 (13.2%)	0.437
Renal failure	8 (4.3%)	14 (3.8%)	0.758
Permanent pacemaker implantation	4 (2.1%)	9 (2.4%)	0.921
Pneumonia	4 (2.2%)	5 (1.3%)	0.722
New onset atrial fibrillation	45 (24.2%)	97 (26.1%)	0.631
IABP	5 (2.7%)	11 (3.0%)	0.858
ECMO	2 (1.1%)	3 (0.8%)	0.874

*CPB* cardiopulmonary bypass, *ICU* intensive care unit, *LOS* length of stay, *IABP* intra-aortic balloon pump, *ECMO* extracorporeal membrane oxygenation

### Comparison between the surgeon’s early and late experience

**Fig. 4:**
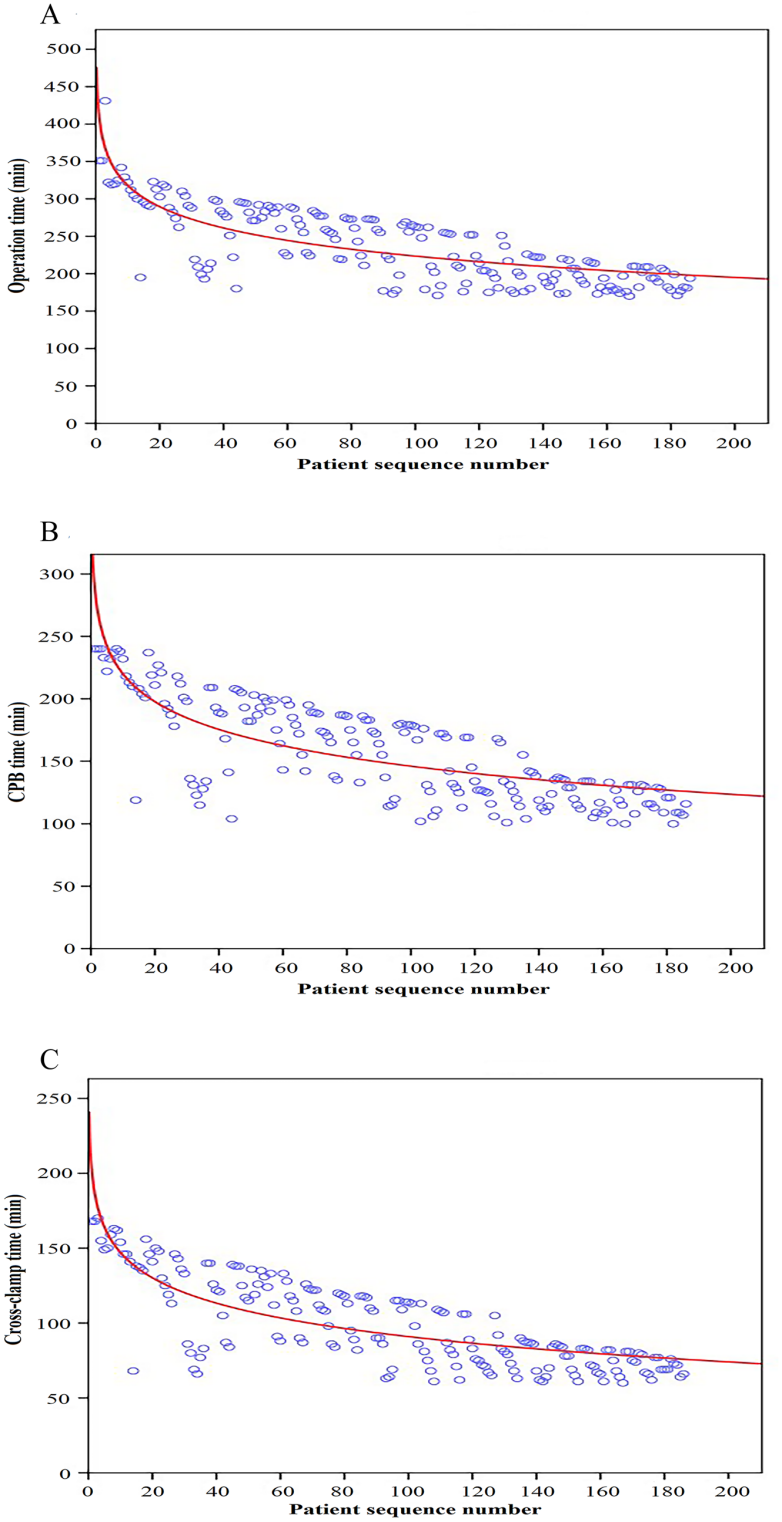
The learning curve of robotic mitral valve replacement. **A** Operation time: y (min) = 412.52x^−41.04^; r^2^ = 0.623; P < 0.001. **B** CPB time: y (min) = 293.96x^−32.13^; r^2^ = 0.603; P < 0.001. **C** Cross-clamp time: y (min) = 203.26x^−24.39^; r^2^ = 0.631; P < 0.001. *CPB* cardiopulmonary bypass

**Fig. 5:**
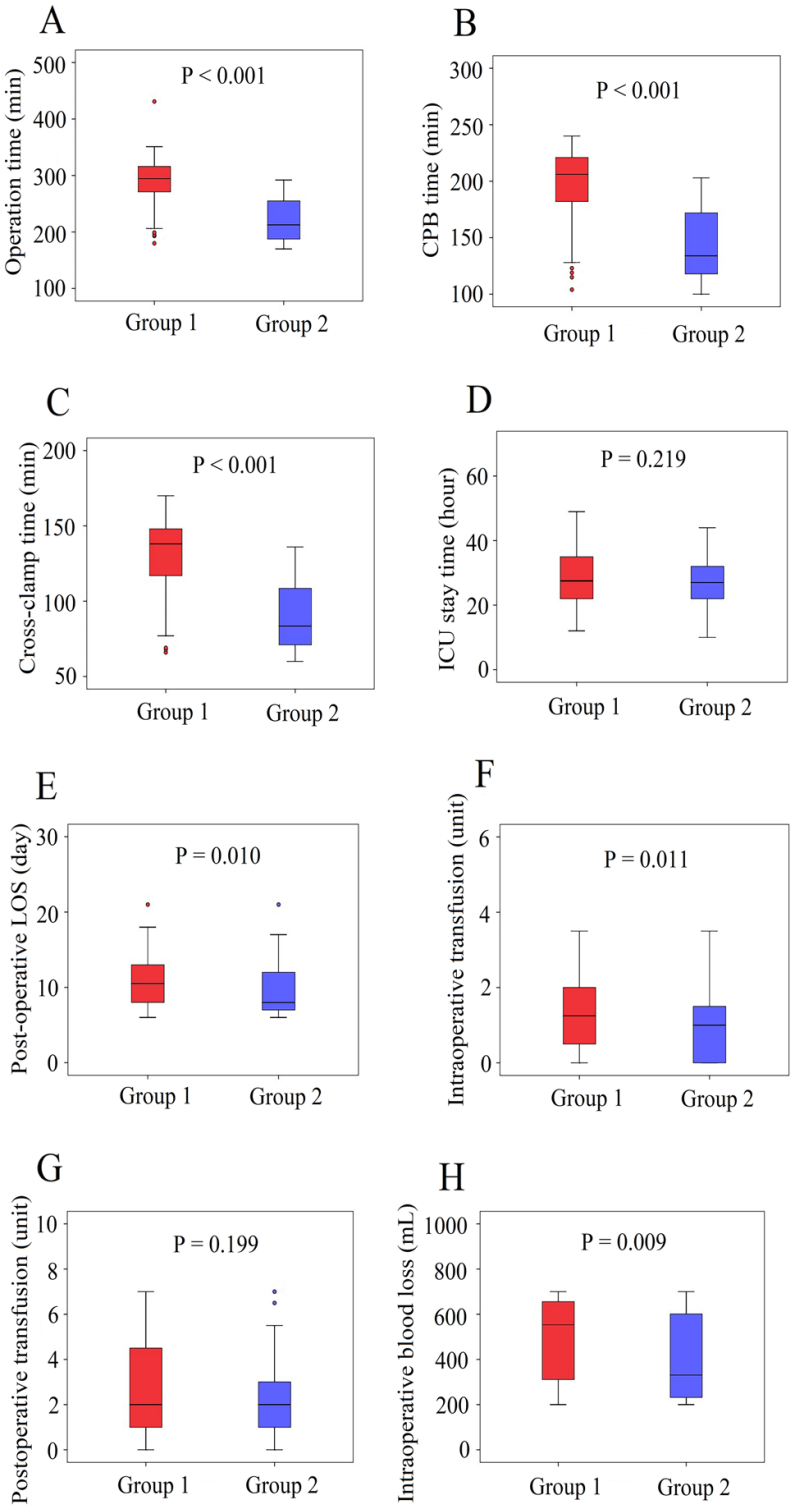
Comparison between the surgeon’s early and late experience of robotic MVR. **A** Operation time. **B** CPB time. **C** Cross clamp time. **D** ICU stay time. **E** Post-operative LOS. **F** Intraoperative transfusion. **G** Postoperative transfusion. H Intraoperative blood loss

The mean operation, CPB and cross-clamp times of robot group were 237 ± 51 min, 155 ± 42 min and 98 ± 31 min, respectively. Moreover, these mean times had a chronological significant improvement with experience (r^2^ = 0.623, P < 0.001; r^2^ = 0.603, P < 0.001; r^2^ = 0.631, P < 0.001, respectively) (Fig. 4). The first 50 cases of robot group were defined as group 1 and the latter 136 cases of robot group were defined as group 2. Compared with group 1, operation time, CPB time, cross-clamp time, post-operative LOS, intraoperative transfusion and intraoperative blood loss were statistically significantly shorter or less in group 2 (Fig. 5).

### Medium-term clinical outcomes

**Fig. 6:**
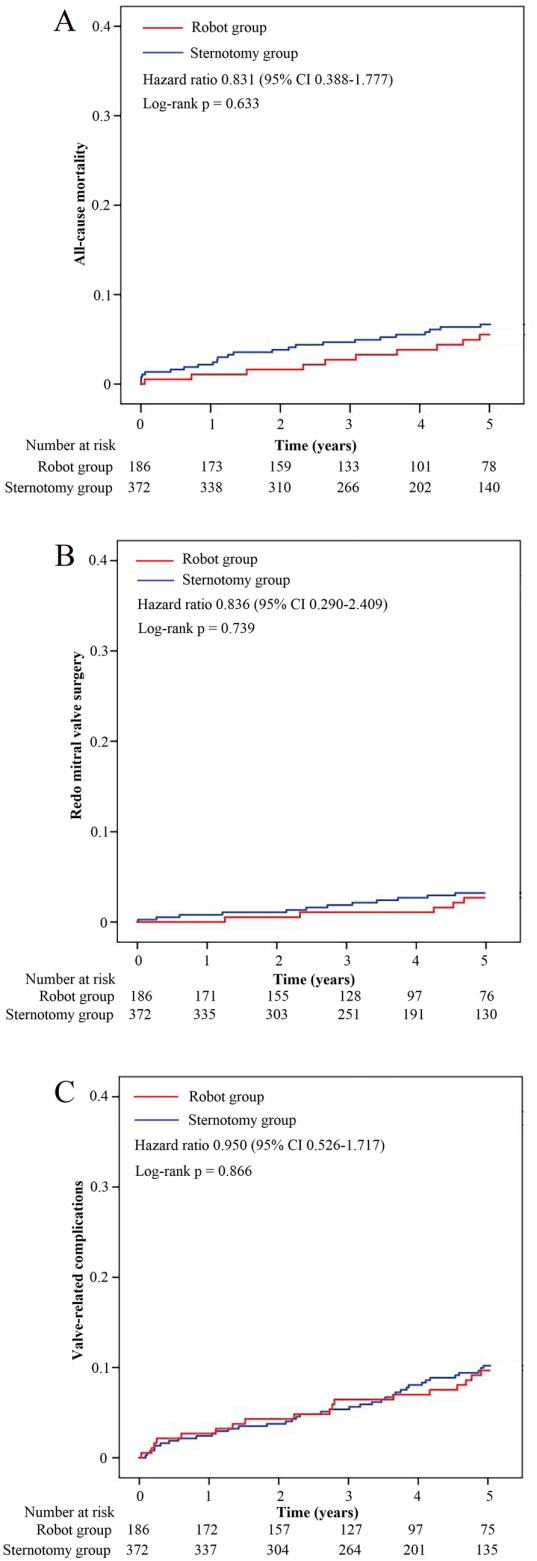
Time-to-event curves for clinical outcomes after matching. **A** All-cause mortality. **B** Redo mitral valve surgery. **C** Valve-related complications

Telephonic and outpatient follow-up appointments were set-up to obtain the clinical data of our population (Tables 6 and 7 and Fig. 6). A total of 546 cases (98%) after matching were successfully followed up, which included 181 cases (97%) in the robot group and 365 cases (98%) in the sternotomy group. All-cause mortality (HR 0.831, 95% CI 0.388–1.777, P = 0.633), redo mitral valve surgery (HR 0.836, 95% CI 0.290–2.409, P = 0.739) and valve-related complications (HR 0.950, 95% CI 0.526–1.717, P = 0.866) were similar between both groups. After matching, 5 patients underwent redo mitral valve surgery at 5-year follow up in the robot group. The reasons were paravalvular leak (n = 1), regurgitation or stenosis (n = 2), endocarditis (n = 2). And 12 patients underwent redo mitral valve surgery at 5-year follow up in the sternotomy group. The reasons were paravalvular leak (n = 3), regurgitation or stenosis (n = 2), endocarditis (n = 3), thrombosis (n = 3) and stuck valve (n = 1).

**Table 6:** Five-year clinical outcomes of robotic MVR vs sternotomy MVR before matching

	Robot group (n = 181)	Sternotomy group (n = 1176)	P value	HR	95% CI
All-cause mortality	10 (5.5%)	99 (8.4%)	0.182	0.636	0.326–1.243
Redo mitral valve surgery	5 (2.8%)	60 (5.1%)	0.170	0.528	0.209–1.334
Valve-related complications	18 (9.9%)	137 (11.6%)	0.502	0.837	0.499–1.406
Bleeding events	9 (5.0%)	66 (5.6%)	0.726	0.880	0.431–1.798
Thromboembolic events	8 (4.4%)	53 (4.5%)	0.958	0.980	0.458–2.096
Infective endocarditis	2 (1.1%)	21 (1.8%)	0.725	0.615	0.143–2.643

**Table 7:** Five-year clinical outcomes of robotic MVR vs sternotomy MVR after matching

	Robot group (n = 181)	Sternotomy group (n = 365)	P value	HR	95% CI
All-cause mortality	10 (5.4%)	24 (6.5%)	0.633	0.831	0.388–1.777
Redo mitral valve surgery	5 (2.7%)	12 (3.2%)	0.739	0.836	0.290–2.409
Valve-related complications	18 (9.7%)	38 (10.2%)	0.866	0.950	0.526–1.717
Bleeding events	9 (4.8%)	20 (5.4%)	0.804	0.903	0.402–2.024
Thromboembolic events	8 (4.3%)	14 (3.8%)	0.744	1.159	0.477–2.816
Infective endocarditis	2 (1.1%)	5 (1.3%)	1.000	0.804	0.155–4.187

## Discussion

In the current propensity score matched study, it was found that the robot group had prolonged procedural times but shorter ICU stay time and post-operative LOS than the sternotomy group. Moreover, the rate of perioperative and medium-term clinical complications was similar between the robot and sternotomy groups. During follow-up, all-cause mortality, redo mitral valve surgery and valve-related complications were similar between the two groups. We also observed a statistically significant reduction in procedure-related times on the learning curve in the robot group.

For severe mitral valve disease, surgery is the recommended treatment. Mitral valve repair is the preferred option. When repair is not available, biological or mechanical mitral valve replacement can be performed. Pettinari et al. [10] have reported a resurgence of interest in robotic mitral valve surgery, which is becoming more mature due to technological advances. Mounting publications demonstrate a remarkable improvement in pain intensity and recovery times correlated with robotic mitral valve surgery. Gillinov et al. [11]. analyzed the outcomes of 1,000 robotic mitral valve surgeries at their center, they found that the robotic mitral valve surgery group had a higher likelihood of valve repair and lower operative mortality and morbidity. Arghami and colleagues [12] used echocardiography for a prolonged follow-up of 843 cases having robotic mitral valve repair, and showed that absent need of reoperation and survival were 92.6% and 93% at 10 years, respectively, indicating the effectiveness and safety of this modality with favorable prolonged outcomes that are not inferior to sternotomy.

Although the effectiveness of robotic MV surgery is widely recognized, previous studies mainly focus on mitral valve repair surgery [6–8, 11–14], and few studies independently verified the effect of robotic mitral valve replacement surgery. Robotic mitral valve replacement is more demanding and complex than repair. Arranging valve sutures and implanting prostheses through a small surgical wound might be the main challenges for conducting this modality. In order to solve these two difficulties, we adjusted the working port in the surgery (Fig. 2A). An enlarged 4-5 cm working port is made in the right fourth intercostal space, lateral to the anterior axillary line. Chitwood Transthoracic Aortic Cross-clamp, cardioplegic solution and robotic camera were administered directly through the working port. This adjustment of the working port made it easier to cross-clamp the aorta, increased the range of motion of the robotic camera, and provided a better surgical view, while reducing the difficulty of valve suture. In our series, the mean operation, CPB, and cross-clamp times of robot group were 237, 155 and 98 min, respectively. This result is better than some related studies [15, 16]. One reason is the adjustment we made to the working port, another reason may be that our robotic surgery was performed by a team which including fixed surgeons, anesthesiologists and nurses. Robot heart surgery requires excellent team cooperation. Figure 4 shows our learning curve, and we observed a trend that the mean operation, CPB, and cross-clamp times all decreased with experience on the learning curve. Further, we compared the perioperative parameters of the first 50 cases and the latter 136 cases in the robot group. Compared with the first 50 cases, operation time, CPB time, cross-clamp time, post-operative LOS, intraoperative transfusion and intraoperative blood loss were statistically significantly shorter or less in the latter 136 cases. Optimal outcomes can be attained with robotic MVR, as shown by our experience, but only after a significant learning curve and with a competent robotic crew.

Differences between the two groups have been accounted for using robust statistical approaches, and there is a satisfactory match for all measurable confounders (Fig. 3). To reflect real-world conditions, mitral combined with tricuspid repair, radiofrequency ablation, and thrombectomy were included in this study and included as matching factors in Tables 2 and 3. The operating death rate in our group is significantly reduced more than the predicted mortality based on STS PROM, indicating good operative results. Within propensity matched cohorts, although operation, CPB, and cross-clamp times were still longer in the robot group than the thoracotomy group, there was no difference in operative mortality, permanent stroke, renal failure and other complications. Furthermore, ICU stay time and post-operative LOS were statistically significantly shorter in the robot group. There are some complications that are inherent in perfusion and ventilation methods used for robotic surgery [5, 9, 17, 18]. Possible major complications related to retrograde cardiopulmonary perfusion modalities include unilateral pulmonary edema and pneumonia, prolonged ventilation, and stroke. However, in our cohort, no differences in these complications were found between the two groups either before or after matching. We hypothesize that the excellent operative outcomes in our cohort are due to the strict screening criteria before surgery. The objective of robotic MVR is to reduce patient recovery time, improve incision-related complications, improving cosmesis and operative outcomes. The anticipated favorable outcomes should not outweigh the medium-term or longer-term surgical outcomes. The current findings are supported by similar investigations in the literature [19, 20] indicating the similarity between both modalities regarding medium-term clinical outcomes including all-cause mortality, redo mitral valve surgery and valve-related complications.

There are some limitations with this study. First, although PSM was done, confounding differences between the two treatment groups could not be completely ruled out due to the nature of the retrospective study. Second, only cases with irreparable MV disease had valve replacement surgeries, decreasing our sample size. Third, although the robot group was performed by the same team, the thoracotomy group was performed by multiple teams, so there may be a potential surgeon bias.

In conclusion, robotic MVR is safe, feasible and reproducible with good operative outcomes and medium-term clinical outcomes. Robotic MVR was associated with longer operation, CPB and cross-clamp times. Whereas ICU stay time, post-operative LOS, intraoperative transfusion and intraoperative blood loss were shorter or less in the robot group. And operation, CPB and cross-clamp times can all improve remarkably with experience. Cases requiring MVR should be aware about the potential to receive a robotic surgery.

## Data Availability

Data available upon request.
